# Supplementary material for: De novo emergence of a remdesivir resistance mutation during treatment of persistent SARS-CoV-2 infection in an immunocompromised patient: a case report
Source: Nat Commun. 2022 Mar 17;13:1547. doi: 10.1038/s41467-022-29104-y (PMC8930970; doi:10.1038/s41467-022-29104-y)
Supplement: Supplementary file 1 — Supplementary information [file 41467_2022_29104_MOESM1_ESM.pdf]

Supplementary Information: De novo emergence of a remdesivir resistance mutation during treatment of persistent SARS-CoV-2 infection in an immunocompromised patient: A case report

S. Gandhi et al

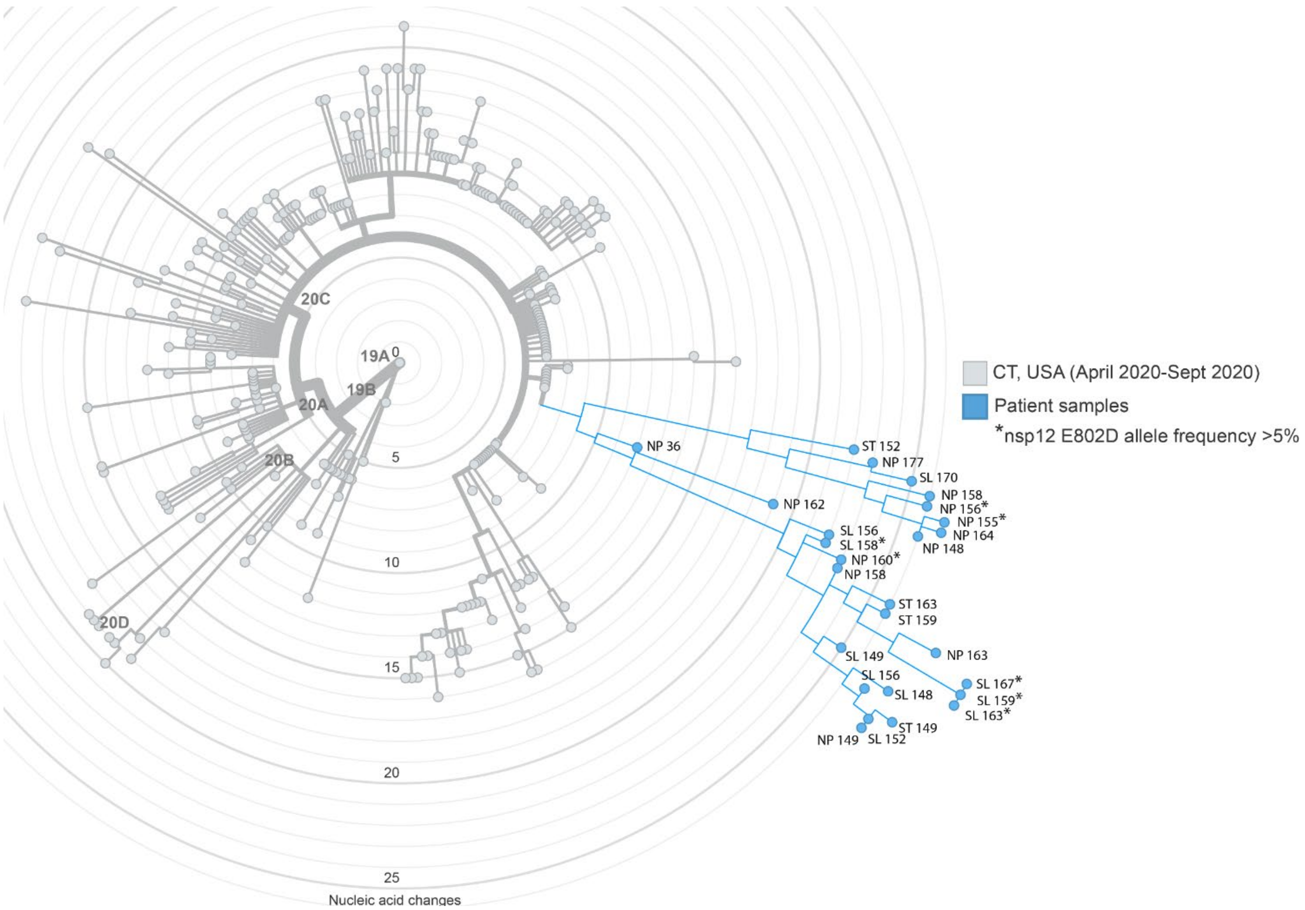

Supplementary Figure 1: Phylogenetic analysis of SARS-CoV-2 genomes from patient samples and contemporaneous samples from Connecticut, USA.

Nextstrain maximum likelihood analysis of patient whole genome samples (blue) in comparison to 283 contemporaneous local samples (grey) obtained from GISAID (10/30/21; Supplemental Table 3). Samples are annotated by type (nasopharyngeal (NP), saliva (SL), or stool (ST)) and day from diagnosis. Asterisk denotes consensus genomes that contain nsp12 E802D. Divergence from root reference genome (Wuhan-Hu-1) by nucleic acid changes.

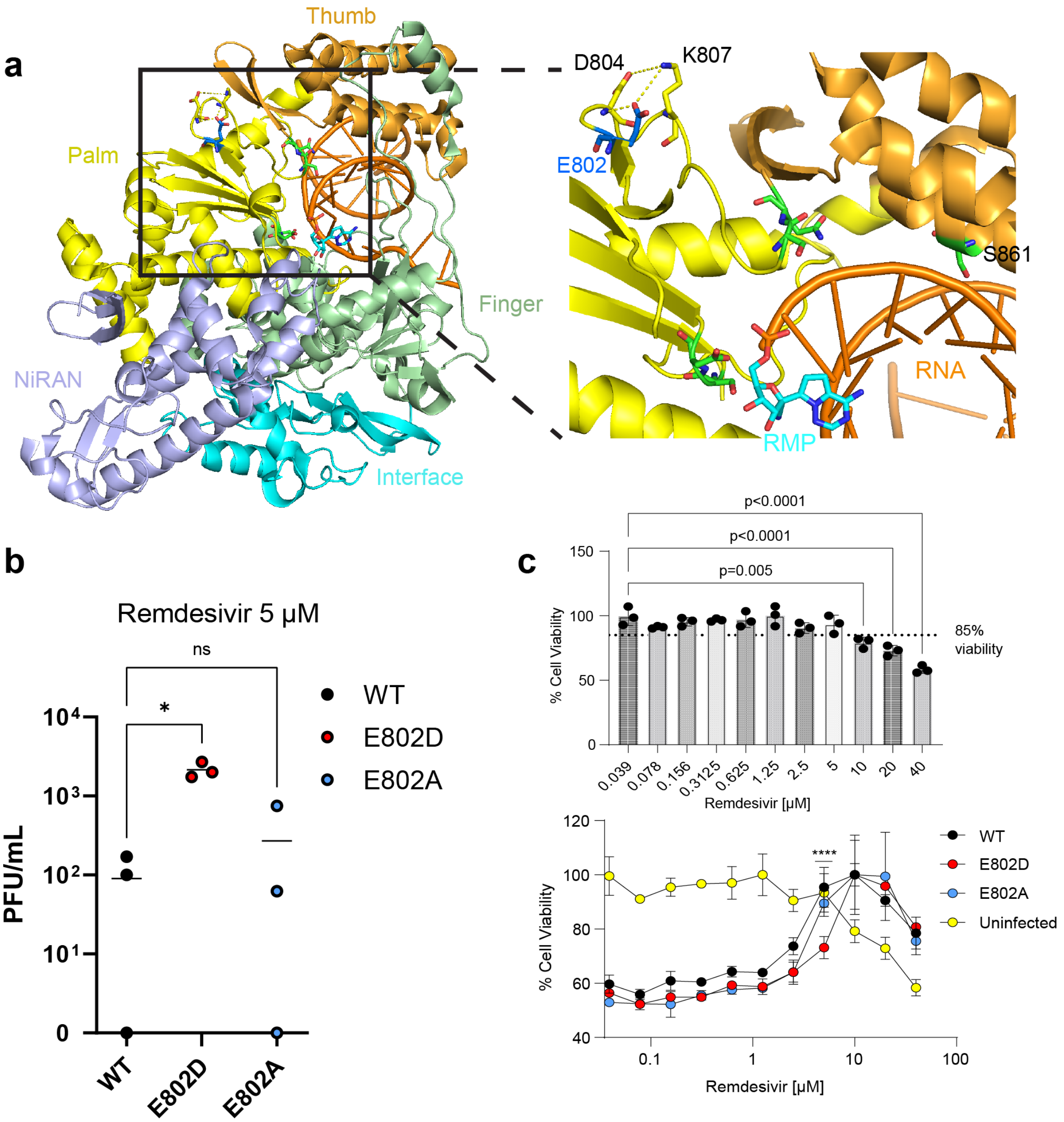

Supplementary Figure 2: Validation of a remdesivir resistance mutation.

a) Structure depicting E802 in relationship to nsp12 (PDB:7BV2) b) Viral titers (PFU) assessed under 5 µM RDV treatment. Biological replicates are depicted (n=3) with the mean represented by the horizontal line. Significance was assessed by an ANOVA, *$p < 0.05$ ($p=0.03$). c) RDV cell viability experiment of uninfected (top panel) and cells infected with the SARS-CoV-2 strains (bottom panel) performed using CellTiter Glo. In the top panel, means and SD of biological replicates (n=3) are represented by bars and error bars, respectively. In the bottom

panel, data is represented as the mean and SD of biological replicates (n=3). Samples were compared using a two-way ANOVA corrected for multiple comparisons. In bottom panel, significance for WT vs E802D is shown, ****$p < 0.0001$.

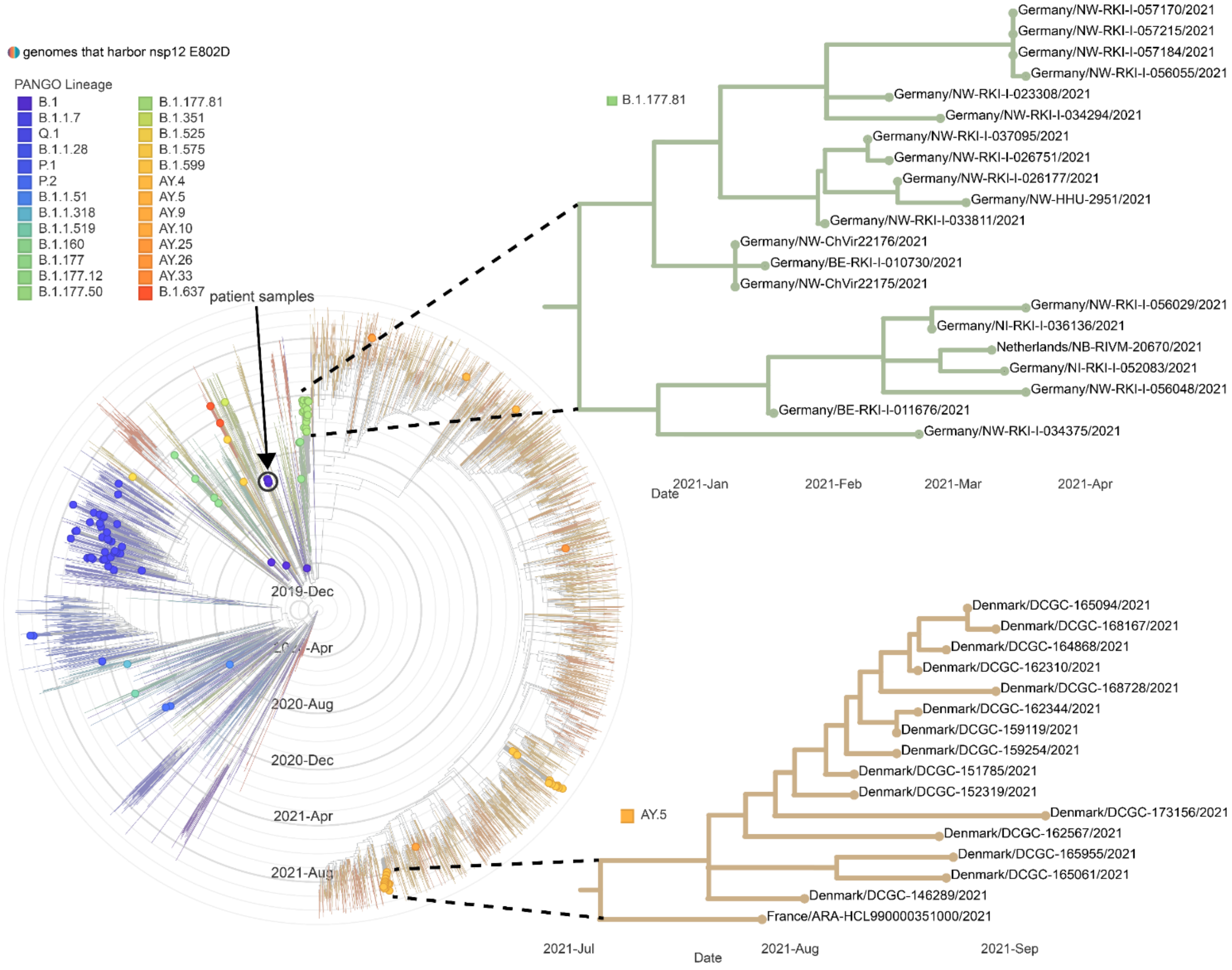

Supplementary Figure 3: Global analysis of genomes with nsp12 E802D mutations.

Nextstrain maximum likelihood analysis of 131 genomes containing the E802D mutation in comparison to the global representative Auspice dataset. Genomes with the E802D mutation (denoted by circles) are distributed on various PANGO lineages (color coded according to the legend). The four patient genomes harboring the mutation are denoted by the arrow. Two genetically, geographically, and temporally linked networks are depicted in detail.

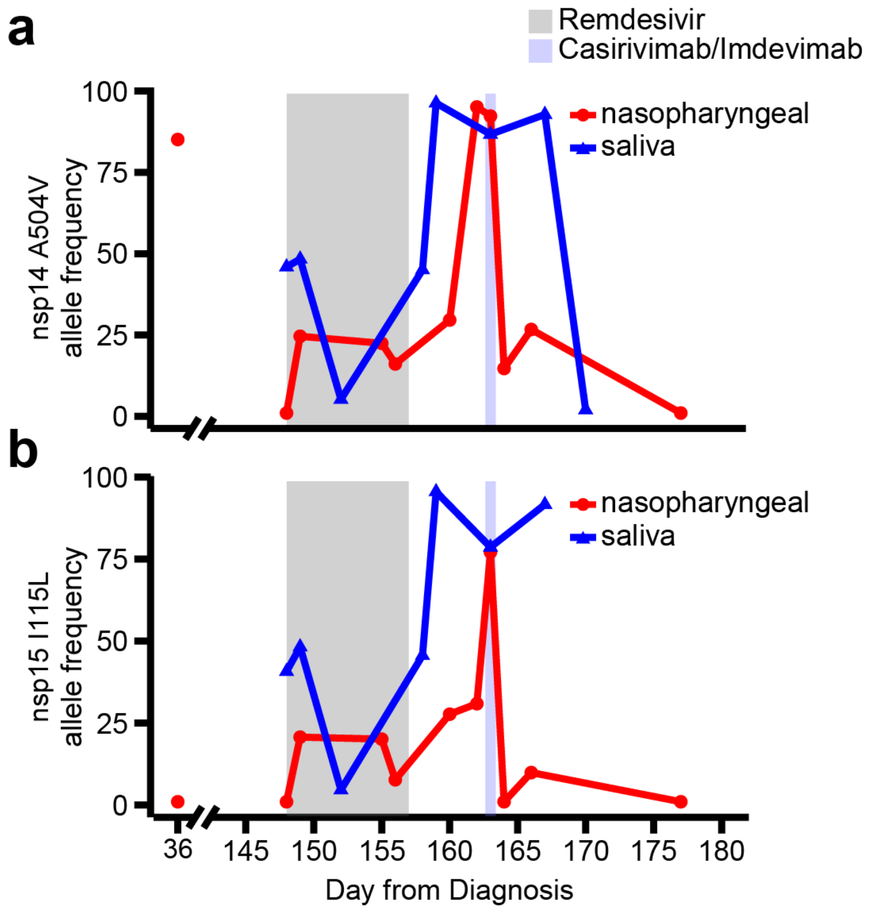

Supplementary Figure 4: Identification of additional mutations that were temporally associated with the initiation of remdesivir therapy.

Longitudinal allele frequencies of A504V in nsp14 (a) and I115L (b) by whole genome sequencing of in relationship to RDV and casirivimab/imdevimab

SNP	Protein	AA	NP 36	NP 148	SL 148
G521T	leader protein	V86F			x
G569A	leader protein	E102K		x	
C1059T	nsp2	T85I	x	x	x
A4229G	nsp3	T504A		x	
C4230G	nsp3	T504R			x
C5178T	nsp3	T820I	x		x
G5180A	nsp3	D821N		x	
C9344T	nsp4	L264F			x
C9870T	nsp4	T439M		x	
G9929A	nsp4	D459N			x
A10323G	3C-like_proteinase	K90R		x	
G11083T	nsp6	L37F			x
A12519G	nsp8	D143G		x	
C12525T	nsp8	T145I			x
A12759G	nsp9	D25G		x	
A12759C	nsp9	D25A			x
C14408T	RdRp	P323L	x	x	x
C19550T	3'-to-5' exonuclease	A504V	x		
A23403G	surface glycoprotein	D614G	x	x	x
C23525T	surface glycoprotein	H655Y			x
C23997T	surface glycoprotein	P812L		x	
C25511T	ORF3a_protein	S40L			x
G25563T	ORF3a_protein	Q57H	x	x	x
C25714T	ORF3a_protein	L108F		x	
G25912T	ORF3a_protein	G174C		x	
A26162del	ORF3a_protein	N257fs	x	x	x
T26767C	membrane glycoprotein	I82T		x	
A28281G	nucleocapsid	D3G		x	
T28829C	nucleocapsid	S186P			x
C28472A	nucleocapsid	P67T	x	x	x
G29151A	nucleocapsid	R293K	x		
A29567G	ORF10_protein	I4V			x

Supplementary Figure 5: Variants identified in the immunocompromised patient with persistent SARS-CoV-2 infection prior to initiation of remdesivir therapy.

Pre-treatment non-synonymous (NS) mutations identified in consensus genomes at days 36 (NP) and 148 (NP and saliva) of infection. During early infection (day 36), whole genome sequencing identified 9 NS mutations from reference strain Wuhan-1 (NC_045512). Six of those persisted through day 148 (green background, start day of RDV treatment). Abbreviations: single nucleotide polymorphism (SNP); amino acid (AA); nasopharyngeal (NP); saliva (SL)

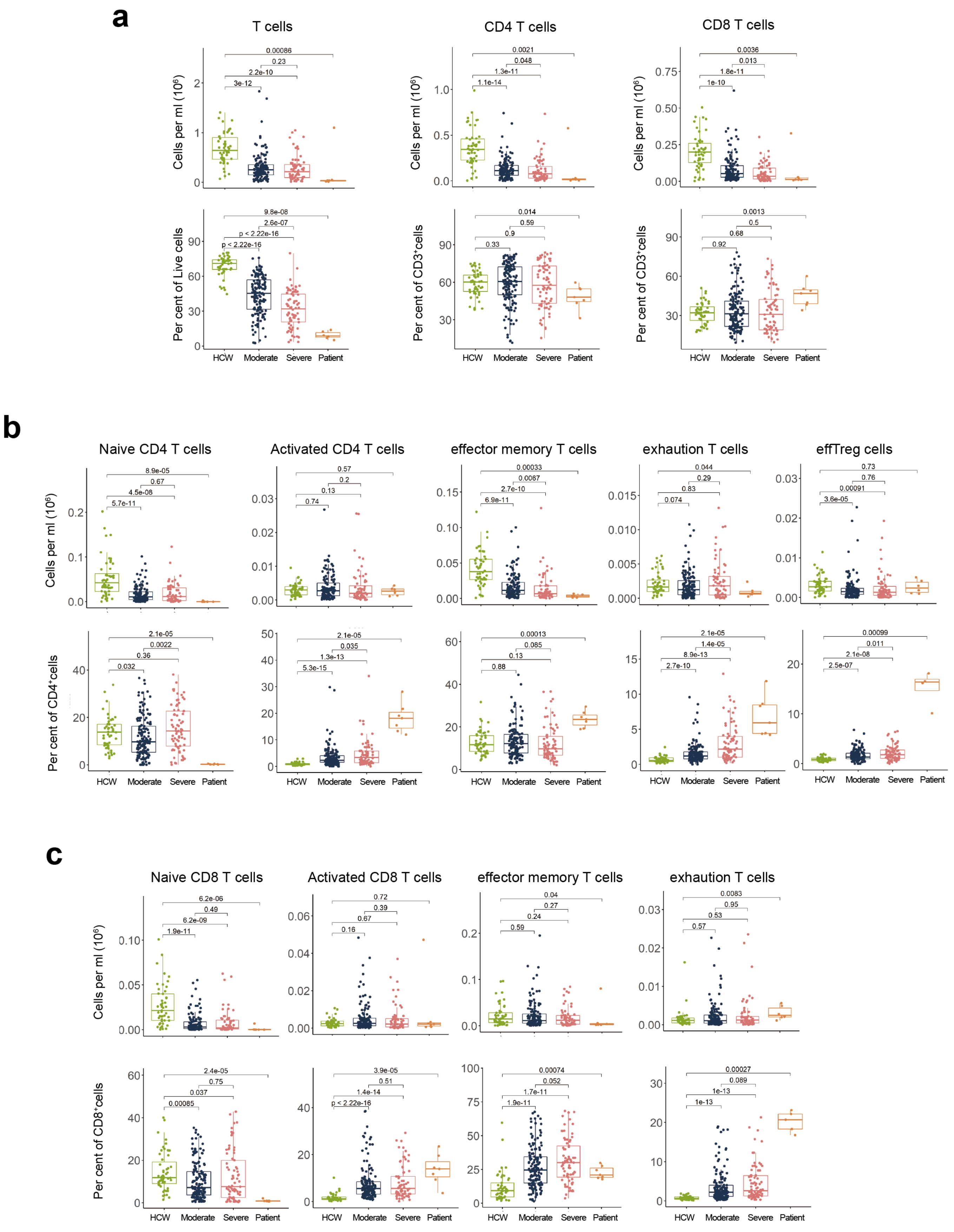
a
T cells
CD4 T cells
CD8 T cells
Cells per ml (10^6)
Per cent of Live cells
Per cent of CD3+cells
HCW
Moderate
Severe
Patient
b
Naive CD4 T cells
Activated CD4 T cells
effector memory T cells
exhaution T cells
effTreg cells
Per cent of CD4+cells
c
Naive CD8 T cells
Activated CD8 T cells
effector memory T cells
exhaution T cells
Per cent of CD8+cells

Supplementary Figure 6: Serial lymphocyte profiling reveals T-cell exhaustion.

T-cell profiling of uninfected healthcare workers (HCW), patient's with moderate and severe SARS-CoV-2 infection. Each point represents a separate time point from each subject (HCW, n=49; moderate, n=118; severe, n=44; patient, n=7). a) Global, $CD4^+$, and $CD8^+$ T-cells. $CD4^+$ (b) and $CD8^+$ (c) subsets demonstrates a phenotype consistent with T-cell exhaustion. For all boxplots, the centre is drawn through the median of the measurement, while the lower and upper bounds of the box correspond to the first and third percentile. Whiskers beyond these points denote 1.5×the interquartile range. P values were determined by two-sided, Wilcoxon rank-sum test. ****p < .0001 ***p < .001**p < .01. *p < .05. Population definitions: naïve CD4/CD8 subset: CD4/CD8+CD45RA+CD127+CCR7+PD-1-; activated CD4/CD8: CD4/CD8+CD38+HLA-DR+;effector memory CD4/CD8:CD4/CD8CD45RA-CD127+CCR7-; exhaustion CD4/CD8: CD4/CD8CD45RA-Tim-3+PD-1+; effTreg: CD4+CD45RA-CD127-CD25hiHLA-DR+. Gating strategy depicted in Fig S8.

a

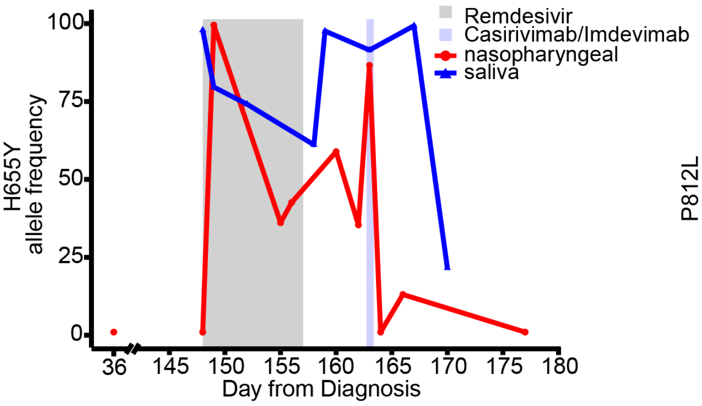

b

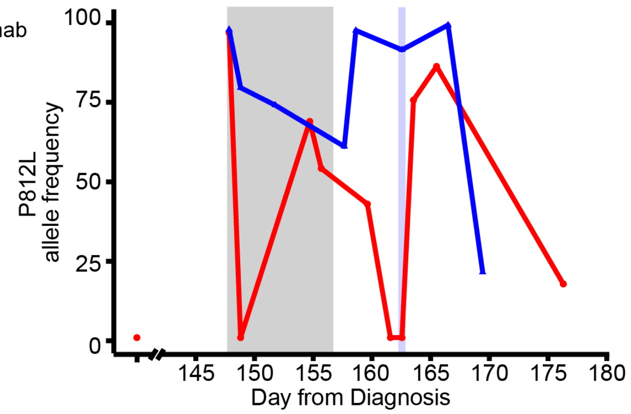

c

Source	Day	D80Y	L141fs	A348S	D614G	V635G	H655Y	V656I	P812L	G1124V
NP	36	<5	<5	<5	100	<5	<5	<5	<5	<5
NP	148	<5	<5	<5	99.9	<5	<5	<5	96.9	<5
SL	148	<5	<5	<5	99.9	<5	97.9	<5	<5	<5
NP	149	<5	<5	<5	99.9	<5	99.5	<5	<5	<5
SL	149	<5	<5	<5	99.9	5.6	79.6	<5	20.7	<5
SL	152	<5	<5	8.8	99.8	<5	74.1	<5	<5	<5
NP	155	<5	<5	<5	99.8	<5	36.1	7.9	69	<5
NP	156	<5	<5	<5	99.8	<5	42.6	<5	54.2	<5
SL	158	<5	<5	<5	99.9	6.2	61.1	<5	20.9	<5
NP	160	<5	<5	<5	99.8	<5	58.9	<5	42.3	<5
NP	162	62.9	<5	<5	99.8	<5	35.4	24.3	<5	65.5
NP	163	13.9	<5	<5	99.8	<5	86.6	59.2	<5	15.8
SL	163	<5	<5	<5	100	5.3	91.5	11.3	10.4	<5
NP	164	17.9	12.6	<5	99.9	<5	<5	<5	75.7	17.3
NP	166	<5	<5	<5	100	<5	13.1	13.2	86.3	8.6
SL	167	<5	<5	<5	100	5.4	99.3	7.4	<5	<5
SL	170	<5	5.6	75	100	5.4	21.6	<5	<5	<5
NP	177	<5	<5	86.5	100	<5	<5	<5	17.9	<5

d

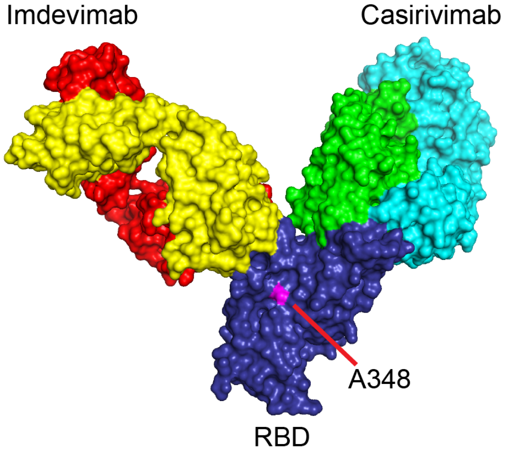

Supplementary Figure 7: Evolution of Spike protein in patient samples.

a) H655Y and b) P812L allele frequency over time. c) Allele frequencies of non-synonymous Spike protein mutations identified on more than one occasion by whole genome sequencing. Allele frequencies >50% are denoted by green background. d) Crystral structure of RBD (PDB:6XDG) depicting residue A348 in relationship to the imdevimab (heavy chain: yellow; light rain: red) and casirivimab (heavy chain: green; light chain: blue) binding sites. Abbreviations: nasopharyngeal (NP); saliva (SL); receptor binding domain (RBD).

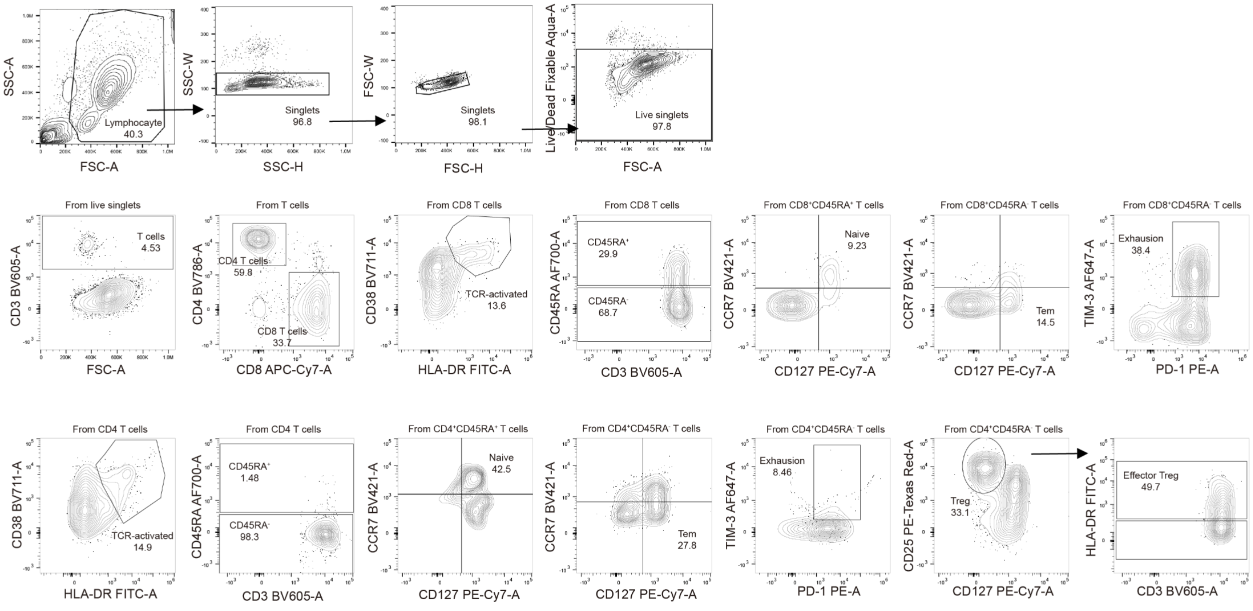

Supplementary Figure 8: Gating strategy for flow cytometry

Gating strategies are shown for the key T cell populations described in Figure S6. The T cell surface staining gating strategy to identify CD8 & CD4 T cells, naïve T cells, TCR-activated T cells, exhaustion T cells, effector memory T cells (Tem), and effector regulatory T cells (eff Treg) are depicted.

Days after diagnosis	Nasopharyngeal specimen N1 RT-PCR Ct	N2 RT-PCR Ct	RP RT-PCR Ct	Viral Culture PFU/ml	Saliva specimen N1 RT-PCR Ct	N2 RT-PCR Ct	RP RT-PCR Ct	Stool specimen N1 RT-PCR Ct	N2 RT-PCR Ct	RP RT-PCR Ct	Whole blood specimen N1 RT-PCR Ct	N2 RT-PCR Ct	RP RT-PCR Ct
36	**21.8**	**21.5**	**-**										
148	**26.1**	**25.4**	**32.9**		**22.9**	**22.2**	**22.3**						
149	**19.2**	**18.0**	**24.0**	**41.7**	**29.4**	**29.8**	**21.8**	**30.0**	**31.6**	**36.6**	ND	ND	36.0
152	**28.3**	**27.7**	**27.8**	**12.5**	**28.9**	**29.0**	**22.1**	**33.2**	**32.9**	**34.4**			
155	**26.1**	**25.6**	**27.6**										
156	**26.2**	**26.1**	**28.2**	**1741.7**	**28.0**	**27.6**	**23.3**	**32.8**	**ND**	**33.7**			
157											ND	ND	26.9
158	**24.2**	**23.7**	**34.5**	**16666.7**	**30.0**	**30.8**	**23.0**	**32.4**	**ND**	**33.1**	ND	ND	27.9
159					**22.1**	**21.2**	**23.5**	**31.7**	**ND**	**36.4**	ND	ND	28.2
160	**23.2**	**22.7**	**27.0**								ND	ND	29.2
162	**23.1**	**22.7**	**28.7**								ND	ND	27.1
163	**22.5**	**22.1**	**28.3**	**533.3**	**27.1**	**27.2**	**22.1**	**32.7**	**33.9**	**34.5**	ND	ND	30.6
164	**27.3**	**27.1**	**28.5**	**0.0**									
166	**35.5**	**36.1**	**29.3**	**0.0**									
167	36.2	36.9	30.7		**28.0**	**27.9**	**23.1**	34.1	41.1	31.5	ND	ND	30.1
168	39.5	38.7	28.2	0.0									
170	36.7	40.2	26.9	0.0	**31.4**	**32.4**	**22.4**	34.1	37.7	33.0	ND	ND	30.0
171	39.4	41.0	30.9										
176	39.7	ND	28.0	0.0	33.9	36.9	22.3	35.0	ND	32.5	ND	ND	29.8
177	**33.3**	**33.7**	**26.2**										
189											ND	ND	28.0
202	ND	ND	33.7								ND	ND	30.3
210	39.8	44.3	29.1	0.0									
217	35.9	37.1	24.4	0.0							ND	ND	28.1
245								34.5	ND	31.9	ND	ND	29.1

Supplementary Table 1: Serial patient samples from various tissues and secretions.

Catalogue of patient samples collected for research during the course of illness. N1, N2, and RP cycle threshold values are reported for each sample. WGS was performed on the bolded samples (accession numbers in table S2). Days of RDV treatment are denoted by grey shading and casirivimab/imdevimab was administered on day 163 (blue shading). Note that NP Ct values depicted in Figures 1,2 were obtained from clinical platforms and are not shown here.

Abbreviations: ND=Not Detected

Sample Name	SRA Accession	GenBank Accession
INP-0411-NP-148	SRR16576689	OL307721
INP-0411-NP-149	SRR16576688	OL307722
INP-0411-NP-152*	SRR16576677	
INP-0411-NP-155	SRR16576668	OL307723
INP-0411-NP-156	SRR16576667	OL307724
INP-0411-NP-158	SRR16576666	OL307725
INP-0411-NP-160	SRR16576665	OL307726
INP-0411-NP-162	SRR16576664	OL307727
INP-0411-NP-163	SRR16576663	OL307728
INP-0411-NP-164	SRR16576662	OL307729
INP-0411-NP-166	SRR16576687	OL307720
INP-0411-NP-177	SRR16576686	OL307730
INP-0411-NP-36	SRR16576685	OL307731
INP-0411-SL-148	SRR16576684	OL307732
INP-0411-SL-149	SRR16576683	OL307733
INP-0411-SL-152	SRR16576682	OL307734
INP-0411-SL-156	SRR16576681	OL307735
INP-0411-SL-158	SRR16576680	OL307736
INP-0411-SL-159	SRR16576679	OL307737
INP-0411-SL-163	SRR16576678	OL307738
INP-0411-SL-167	SRR16576676	OL307739
INP-0411-SL-170	SRR16576675	OL307740
INP-0411-ST-149	SRR16576674	OL307741
INP-0411-ST-152	SRR16576673	OL307742
INP-0411-ST-156	SRR16576672	OL307743
INP-0411-ST-158	SRR16576671	OL307744
INP-0411-ST-159	SRR16576670	OL307745
INP-0411-ST-163	SRR16576669	OL307746

*depth was sufficent to calculate allele frequency at position E802 in nsp12 but coverage was insufficent to generate consenus genome

Supplementary Table 2: Accession numbers for submitted samples.

We gratefully acknowledge the following Authors from the Originating laboratories responsible for obtaining the specimens, as well as the Submitting laboratories where the genome data were generated and shared via GISAID, on which this research is based.

All Submitters of data may be contacted directly via www.gisaid.org

Authors are sorted alphabetically.

Accession ID	Originating Laboratory	Submitting Laboratory	Authors
EPI_ISL_3354875	CT-Dr. Katherine A. Kelley State Public Health Lab	Centers for Disease Control and Prevention Division of Viral Diseases, Pathogen Discovery	Alex Burgin; Ben Rambo-Martin; Clinton Paden; Dakota Howard; Dave Wentworth; Dhwani Batra; Jasmine Padilla; Justin Lee; Krista Queen; Kristen Knipe; Kristine Lacek; Mark Burroughs; Matthew Schmerer; Meghan Bentz; Mili Sheth; Peter Cook; Sam Shepard; Sarah Nobles; Suxiang Tong; Vivien Dugan; Yvette Unoarumhi
EPI_ISL_454642, EPI_ISL_454645	CT-Dr. Katherine A. Kelley State Public Health Lab	Pathogen Discovery, Respiratory Viruses Branch, Division of Viral Diseases, Centers for Disease Control and Prevention	Anna Uehara; Bettina Bankamp; Clinton R. Paden; Haibin Wang; Jing Zhang; Krista Queen; Suxiang Tong; Yan Li; Ying Tao; Zachary Weiner
EPI_ISL_729735, EPI_ISL_729736, EPI_ISL_729741, EPI_ISL_729743, EPI_ISL_729744, EPI_ISL_729745, EPI_ISL_729746, EPI_ISL_729747, EPI_ISL_729748, EPI_ISL_729749, EPI_ISL_729750, EPI_ISL_729752, EPI_ISL_729753, EPI_ISL_729754, EPI_ISL_729755, EPI_ISL_729756, EPI_ISL_729758, EPI_ISL_729759, EPI_ISL_729760, EPI_ISL_729765, EPI_ISL_729772, EPI_ISL_729773, EPI_ISL_729778, EPI_ISL_729779, EPI_ISL_729780, EPI_ISL_729786, EPI_ISL_729789, EPI_ISL_729792			
see above	Connecticut Department of Health	Grubaugh Lab - Yale School of Public Health	Adam Moore; Akiko Iwasaki; Albert Ko; Alice Lu; Allison Nelson; Anderson Brito; Anne Wyllie; Annie Watkins; Arnau Casanovas; Catherine Muenker; Chaney Kalinich; Chantal Vogels; Charlese Dela Cruz; Isabel Ott; Joseph Fauver; Maria Tokuyama; Mary Petrone; Nathan Grubaugh; Patrick Wong; Peiwen Lu; Richard Martinello; Saad Omer; Shelli Farhadian; Tara Alpert
EPI_ISL_527738, EPI_ISL_527761, EPI_ISL_527762, EPI_ISL_527763, EPI_ISL_527764, EPI_ISL_527765, EPI_ISL_527766, EPI_ISL_527767, EPI_ISL_527768, EPI_ISL_527769, EPI_ISL_527770, EPI_ISL_527771, EPI_ISL_527772, EPI_ISL_527773, EPI_ISL_527774, EPI_ISL_527775, EPI_ISL_527776, EPI_ISL_527777, EPI_ISL_527778, EPI_ISL_527779, EPI_ISL_527780, EPI_ISL_527781, EPI_ISL_527782, EPI_ISL_527783, EPI_ISL_527784, EPI_ISL_527785			
see above	Connecticut State Department of Public Health	Grubaugh Lab - Yale School of Public Health	Albert Ko; Anderson Brito; Anthony Muyombwe; Chantal Vogels; Jafar Razeq; Joseph Fauver; Mary Petrone; Nathan Grubaugh; Tara Alpert
EPI_ISL_738284, EPI_ISL_738285, EPI_ISL_738289, EPI_ISL_738298, EPI_ISL_738299, EPI_ISL_738300, EPI_ISL_738301, EPI_ISL_738302, EPI_ISL_738307, EPI_ISL_738308, EPI_ISL_738309, EPI_ISL_738310, EPI_ISL_738311, EPI_ISL_738312, EPI_ISL_1017585, EPI_ISL_1017589, EPI_ISL_1017592, EPI_ISL_1017593, EPI_ISL_1017602, EPI_ISL_1017604, EPI_ISL_1017627, EPI_ISL_1017628, EPI_ISL_1017636, EPI_ISL_1017637, EPI_ISL_1017639, EPI_ISL_1017643, EPI_ISL_1017652, EPI_ISL_1017675, EPI_ISL_1067646, EPI_ISL_1067647, EPI_ISL_1067686, EPI_ISL_1067687, EPI_ISL_1080455, EPI_ISL_1080462, EPI_ISL_1091839, EPI_ISL_1091869, EPI_ISL_1239847			
see above	Connecticut Veterans' Affairs Hospital	Grubaugh Lab - Yale School of Public Health	Chantal Vogels; Danielle Plank; Ellen Foxman; Isabel Ott; Joseph Fauver; Mary Petrone; Nathan Grubaugh; Shaili Gupta; Tara Alpert
EPI_ISL_802136	MSHS Clinical Microbiology Laboratories	MSHS Pathogen Surveillance Program	Adolfo García-Sastre; Adriana van de Guchte; Ajay Obla; Alberto Paniz-Mondolfi; Ana S. Gonzalez-Reiche; Andrew Kasarskis; Angela Amoako; Ashley S. Salimbangon; Betsaida Salom Melo; Bremy Alburquerque; Brianne Ciferri; Charles Gleason; Deena R. Altman; Denise Jurczyszak; Elena Hirsch; Emilia Mia Sordillo; Emily Ferreri; Gintaras Deikus; Giulio Kleiner; Gopi Patel; Hala Alshammary; Harm van Bakel; Irina Oussenko; Jayeeta Dutta; Juan Soto; Katherine Beach; Kathryn Twyman; Kayla Russo; Komal Srivastava; Levy Sominsky; Mahmoud Awawda; Marta Luksza; Matthew M. Hernandez; Melissa Gitman; Michael D. Nowak; Mitchell J. Sullivan; Nancy Francoeur; Rachel Chernet; Robert Sebra; Sarah Schaefer; Shelcie Fabre; Shwetha Hara Sridhar; Viviana Simon; Ying-Chih Wang; Zenab Khan
EPI_ISL_430401	NYU Langone Health	Departments of Pathology and Medicine, New York University School of Medicine	Adriana Heguy; Amy Rapkiewicz; Andre M. Ribeiro-dos-Santos; Andrew Lytle; Antonio Serrano; Brendan Belovarac; Christian Marier; Dacia Dimartino; Emily Guzman; Emily Huang; Gael Westby; George Jour; Guomiao Shen; Iman Osman; Jared Pinnell; John Cadley; John Chen; Lawrence H. Lin; Ludovic Boytard; Margaret Black; Maria Aguero-Rosenfeld; Marie Samanovic-Golden; Mark J. Mulligan; Matija Snuderl; Matthew T. Maurano; Megan Hogan; Nick Vulpescu; Paolo Cotzia; Paul Zappile; Peter Meyn; Raquel Ordonez Ciriza; Raven Luther; Sitharam Ramaswami; Tatyana Gindin; Theodore Vougiouklakis; Vanessa Raabe; Xiaojun Feng; Yutong Zhang
EPI_ISL_937036, EPI_ISL_937037	Quest Diagnostics	Quest Diagnostics	Anderson, B.; D.F.; Gerasimova, A.; Hua, M.; K.E.; Kagan; Lacbawan, F.; Liu Y.; Livingston; Owen, R.; R.M.; Rosenthal; S.H.; Shalhout
EPI_ISL_429640, EPI_ISL_429641, EPI_ISL_429642, EPI_ISL_429645, EPI_ISL_429646, EPI_ISL_430907, EPI_ISL_430908, EPI_ISL_430917, EPI_ISL_430919, EPI_ISL_430921, EPI_ISL_430922, EPI_ISL_430923, EPI_ISL_430925, EPI_ISL_430926, EPI_ISL_430927, EPI_ISL_430960, EPI_ISL_430961, EPI_ISL_430962, EPI_ISL_430963, EPI_ISL_430965, EPI_ISL_430971, EPI_ISL_430972, EPI_ISL_430973, EPI_ISL_437865, EPI_ISL_437866, EPI_ISL_437867, EPI_ISL_437868, EPI_ISL_437869, EPI_ISL_437870, EPI_ISL_437871, EPI_ISL_437872, EPI_ISL_461408, EPI_ISL_461409, EPI_ISL_461411, EPI_ISL_461412, EPI_ISL_461414, EPI_ISL_461416, EPI_ISL_461417, EPI_ISL_461420, EPI_ISL_461435, EPI_ISL_461441, EPI_ISL_570070, EPI_ISL_570076, EPI_ISL_570092, EPI_ISL_570093, EPI_ISL_570094, EPI_ISL_570095, EPI_ISL_570104, EPI_ISL_570107, EPI_ISL_570109, EPI_ISL_570130, EPI_ISL_570131, EPI_ISL_570132, EPI_ISL_570133, EPI_ISL_570135, EPI_ISL_570137, EPI_ISL_570141, EPI_ISL_570145, EPI_ISL_570146, EPI_ISL_570148, EPI_ISL_570166, EPI_ISL_570178, EPI_ISL_570179, EPI_ISL_570180, EPI_ISL_570181, EPI_ISL_570182, EPI_ISL_570184, EPI_ISL_570185			
see above	UW Virology Lab	UW Virology Lab	Alexander Greninger; Amin Addetia; Hong Xie; Keith Jerome; Keith R Jerome; Lasata Shrestha; Meei-Li Huang; Pavitra Roychoudhury; Truong Nguyen; Victoria M Rachleff
EPI_ISL_484885	University of Wisconsin-Madison AIDS Vaccine Research Laboratories	University of Wisconsin-Madison AIDS Vaccine Research Laboratories	Gage Moreno; Katarina Braun; et al. AIDS Vaccine Research Laboratories
EPI_ISL_1168354, EPI_ISL_1168356, EPI_ISL_1168357, EPI_ISL_1168363, EPI_ISL_1168368, EPI_ISL_1168377, EPI_ISL_1168384, EPI_ISL_1168391, EPI_ISL_1168396, EPI_ISL_1168406, EPI_ISL_1168426, EPI_ISL_1168427, EPI_ISL_1168428, EPI_ISL_1168431, EPI_ISL_1168435, EPI_ISL_1168437			
see above	VA Connecticut Healthcare System	Grubaugh Lab - Yale School of Public Health	Chantal Vogels; Danielle Plank; Isabel Ott; Joseph Fauver; Mary Petrone; Nathan Grubaugh; Shaili Gupta; Tara Alpert
EPI_ISL_428384, EPI_ISL_428385, EPI_ISL_428386, EPI_ISL_428389, EPI_ISL_428391, EPI_ISL_428392, EPI_ISL_428393, EPI_ISL_428394, EPI_ISL_428395, EPI_ISL_428396, EPI_ISL_428397, EPI_ISL_428398, EPI_ISL_428399, EPI_ISL_428401, EPI_ISL_428402, EPI_ISL_428403, EPI_ISL_428404, EPI_ISL_428405, EPI_ISL_428746, EPI_ISL_428747, EPI_ISL_428748, EPI_ISL_428749, EPI_ISL_428750, EPI_ISL_428751, EPI_ISL_428752, EPI_ISL_431081, EPI_ISL_431082, EPI_ISL_431083, EPI_ISL_431084, EPI_ISL_431085, EPI_ISL_431086, EPI_ISL_431087, EPI_ISL_431088, EPI_ISL_431089, EPI_ISL_431090, EPI_ISL_431091, EPI_ISL_431092, EPI_ISL_431093, EPI_ISL_431094, EPI_ISL_431095, EPI_ISL_431096, EPI_ISL_435702, EPI_ISL_435703, EPI_ISL_435704, EPI_ISL_435705, EPI_ISL_435706, EPI_ISL_435707, EPI_ISL_435708, EPI_ISL_452143, EPI_ISL_452144, EPI_ISL_452145, EPI_ISL_452146, EPI_ISL_452147, EPI_ISL_594133, EPI_ISL_730052			
see above	Yale COVID-19 Biorepository	Grubaugh Lab - Yale School of Public Health	Adam Moore; Akiko Iwasaki; Albert Ko; Alice Lu; Allison Nelson; Anderson Brito; Anne Wyllie; Annie Watkins; Arnau Casanovas; Catherine Muenker; Chaney Kalinich; Chantal Vogels; Charlese Dela Cruz; Cole Jensen; Isabel Ott; Joseph Fauver; Maria Tokuyama; Mary Petrone; Nathan Grubaugh; Patrick Wong; Peiwen Lu; Richard Martinello; Saad Omer; Shelli Farhadian; Tara Alpert
EPI_ISL_463167, EPI_ISL_463168, EPI_ISL_463169, EPI_ISL_463170, EPI_ISL_463171, EPI_ISL_463172, EPI_ISL_463173, EPI_ISL_463174, EPI_ISL_469171, EPI_ISL_469172, EPI_ISL_469173, EPI_ISL_469174, EPI_ISL_469175, EPI_ISL_469176, EPI_ISL_469177, EPI_ISL_469178, EPI_ISL_469179, EPI_ISL_469180, EPI_ISL_469181, EPI_ISL_469182, EPI_ISL_469183, EPI_ISL_469184, EPI_ISL_469185, EPI_ISL_469186, EPI_ISL_469187, EPI_ISL_469188, EPI_ISL_469189, EPI_ISL_469190, EPI_ISL_469191, EPI_ISL_469192, EPI_ISL_469193, EPI_ISL_469194, EPI_ISL_469195, EPI_ISL_469196, EPI_ISL_469197, EPI_ISL_469198, EPI_ISL_469199, EPI_ISL_469200, EPI_ISL_469201, EPI_ISL_469202, EPI_ISL_469203, EPI_ISL_469204, EPI_ISL_469205, EPI_ISL_469206, EPI_ISL_469207, EPI_ISL_469208, EPI_ISL_476547, EPI_ISL_476548, EPI_ISL_476549, EPI_ISL_476550, EPI_ISL_476551, EPI_ISL_476552, EPI_ISL_476553, EPI_ISL_476554, EPI_ISL_476555, EPI_ISL_476556, EPI_ISL_476557, EPI_ISL_730062, EPI_ISL_730063, EPI_ISL_730064, EPI_ISL_730065, EPI_ISL_730066, EPI_ISL_730067, EPI_ISL_730068, EPI_ISL_730069, EPI_ISL_730070, EPI_ISL_730071, EPI_ISL_730072, EPI_ISL_730073, EPI_ISL_730074, EPI_ISL_730075, EPI_ISL_730076, EPI_ISL_730077, EPI_ISL_730078, EPI_ISL_730079, EPI_ISL_730080, EPI_ISL_730081, EPI_ISL_730082, EPI_ISL_730083, EPI_ISL_730084			
see above	Yale Clinical Virology Laboratory	Grubaugh Lab - Yale School of Public Health	Adam Moore; Akiko Iwasaki; Albert Ko; Alice Lu; Allison Nelson; Anderson Brito; Anne Wyllie; Annie Watkins; Arnau Casanovas; Catherine Muenker; Chaney Kalinich; Chantal Vogels; Charlese Dela Cruz; Cole Jensen; Isabel Ott; Joseph Fauver; Maria Tokuyama; Mary Petrone; Nathan Grubaugh; Patrick Wong; Peiwen Lu; Richard Martinello; Saad Omer; Shelli Farhadian; Tara Alpert
EPI_ISL_738290, EPI_ISL_738291, EPI_ISL_738292, EPI_ISL_738293, EPI_ISL_738294, EPI_ISL_738295, EPI_ISL_738296, EPI_ISL_738297			
see above	Yale Pathology Lab	Grubaugh Lab - Yale School of Public Health	Chantal Vogels; Danielle Plank; Ellen Foxman; Isabel Ott; Joseph Fauver; Mary Petrone; Nathan Grubaugh; Shaili Gupta; Tara Alpert

Supplementary Table 3: GISAID acknowledgments for local genomes used to generate Supplementary Figure 1

We gratefully acknowledge the following Authors from the Originating laboratories responsible for obtaining the specimens, as well as the Submitting laboratories where the genome data were generated and shared via GISAID, on which this research is based.

All Submitters of data may be contacted directly via www.gisaid.org

Authors are sorted alphabetically.

Accession ID	Originating Laboratory	Submitting Laboratory	Authors
EPI_ISL_4361721, EPI_ISL_4362029	Aegis Sciences Corporation	Centers for Disease Control and Prevention Division of Viral Diseases, Pathogen Discovery	Alec Vest; Benjamin Rambo-Martin; Christopher Gulvick; Clinton Paden; Cyndi Clark; Dakota Howard; Dhwani Batra; Dillon Nall; Duncan MacCannell; Erisa Sula; Ethan Sanders; Holly Houdeshell; Jason Caravas; Kristine Lacek; Matthew Hardison; Matthew Schmerer; Ola Kvalvaag; Patrick Campbell; Peter Cook; Rob Case; Scott Sammons; Shatavia Morrison; Shaun Westlund; Tymeckia Kendall; Victoria Caban Figueroa; Vikramsinha Ghorpade; Yvette Unoarumhi
EPI_ISL_2102156	CHUV	Laboratory of genomics and metagenomics	Claire Bertelli; Damien Jacot; Gilbert Greub; Sébastien Aeby; Trestan Pillonel
EPI_ISL_3473275	CNR Virus des Infections Respiratoires - France SUD	CNR Virus des Infections Respiratoires - France SUD	Antonin Bal; Bruno Lina; Gregory Destras; Gwendolyne Burfin; Hadrien Regue; Laurence Josset; Martine Valette; Quentin Semanas
EPI_ISL_2170967	CS DE NIPOA	Instituto Butantan / Mendelics	Antonio Jorge Martins; Bianca Cechetto Carlos. Mendelics: Bibiana Santos; Claudia Renata dos Santos Barros; Cíntia Bittar; David Schlesinger. Hemocentro Ribeirão Preto: Simone Kashima; Debora Botequio Moretti; Elaine Cristina Marqueze; Elaine Vieira dos Santos; Elisangela Chicaroni Mattos; Erika Freitas; Evandra Strazza Rodrigues; Felipe Allan da Silva da Costa; Flavia Aburjaile; Fábio Sossai Possebon; Guilherme Campos; Guilherme Targino Valente; Heidge Fukumasu. USP-Botucatu: Rejane Maria Tommasini Grotto; Helena Lage Ferreira; Instituto Butantan: Dimas Tadeu Covas; Jardelina de Souza Todao Bernardino; Jayme A. Souza-Neto; Jessika Cristina Chagas Lesbon; Jorge A. Petroli Marchesi; José Salvatore Leister Patané; João Paulo Kitajima; João Pessoa Araújo Jr.; Leila Sabrina Ullmann; Loyze Paola Oliveira de Lima; Luiz Aurelio de Campos Crispin. Centro de Genômica Funcional da ESALQ: Luiz Lehmann Coutinho; Luiz Carlos Junior de Alcantara; Lívia Sacchetto; Maisa C. Pereira Parra; Maria Carolina Elias; Marta Giovanetti; Marília Moraes; Maurício Lacerda Nogueira. Prefeitura de Sao Paulo: Melissa Palmieri.; Patricia Akemi Assato; Paula Rahal; Paulo Inacio da Costa; Rafael dos Santos Bezerra; Raquel de Lello Rocha Campos Cassano. NGS Soluções Genômicas: Pilar Drummond Sampaio Corrêa Mariani. FZEA-USP Pirassununga: Mirele Daiana Poleti; Raul Machado Neto; Ricardo Augusto Brassaloti; Ricardo Haddad; Rodrigo Tocantins Calado. FAMERP-SJRP: Cecília Artico Banho; Sandra Coccuzzo Sampaio; Svetoslav Nanev Slavov; Vagner Fonseca; Vincent Louis Viala
EPI_ISL_1138787	Centro Hospitalar do Baixo Vouga (CHBV)	Institute of Biomedicine (IBiMED), Universidade de Aveiro	Gabriela Moura; Miguel Pinheiro and Manuel Santos; Patricia Arinto; Sofia Marques
EPI_ISL_862176, EPI_ISL_862177	Charité Universitätsmedizin Berlin, Institut für Virologie/Labor Berlin	Charité Universitätsmedizin Berlin, Institut für Virologie	Barbara Mühlemann; Christian Drosten; Julia Schneider; Julia Tesch; Jörn Beheim-Schwarzbach; Talitha Veith; Terry Jones; Tobias Bleicker; Victor M Corman
EPI_ISL_1156362	Chemische und Veterinäruntersuchungsamt Münsterland-Emscher-Lippe	Robert Koch Institute	
EPI_ISL_3339857, EPI_ISL_3463152, EPI_ISL_3463851, EPI_ISL_3707915, EPI_ISL_3708049, EPI_ISL_3770479, EPI_ISL_3770513, EPI_ISL_3806078, EPI_ISL_3904028, EPI_ISL_3904237, EPI_ISL_3904270, EPI_ISL_3917497, EPI_ISL_4007212, EPI_ISL_4008134, EPI_ISL_4355696			
see above	Department of Bacteria, Parasites and Fungi, Statens Serum Institut, Copenhagen, Denmark	Statens Serum Institut Bioinformatics and Microbial Genomics	Danish Covid-19 Genome Consortium
EPI_ISL_1654424	Department of Public Health Microbiology Ljubljana, National Laboratory for Health, Environment and Food	Department for Public Health Microbiology Ljubljana, National Laboratory for Health, Environment and Food	José Gonçalves; Katarina Prosenc; Martin Bosilj; Metka Paragi; Natasa Berginc; Tom Koritnik
EPI_ISL_759609, EPI_ISL_793664	Department of Virus and Microbiological Special Diagnostics, Statens Serum Institut, Copenhagen, Denmark	Albertsen Lab, Department of Chemistry and Bioscience, Aalborg University, Denmark	Danish Covid-19 Genome Consortium
EPI_ISL_666605	Dept. of Microbiology and Infection Control, Akershus University Hospital HF	Dept. of Microbiology and Infection Control, Akershus University Hospital HF	Alexander Hesselberg Løvestad; Hege Vangstein Aamot; Nina Handal; Ole Herman Ambur; Silje Bakken Jørgensen
EPI_ISL_1457742, EPI_ISL_2672226, EPI_ISL_3257488	Dutch COVID-19 response team	National Institute for Public Health and the Environment (RIVM)	Adam Meijer; AnneMarie van den Brandt; Annelies Kroneman; Bas van der Veer; Chantal Reusken; Dennis Schmitz; Dirk Eggink; Euníce Then; Florian Zwagemaker; Harry Vennema; James Groot; Jeroen Cremer; Jolienke Hardeman; Karim Hajji; Kim Freriks; Linda van de Nes; Lisa Wijsman; Lynn Aarts; Melissa van Tuil; Robert Kohl; Ryanne Jaarsma; Sanne Bos; Sharon van den Brink; Sjoerd Kuiling; on behalf of the national COVID-19 response team
EPI_ISL_4636562	Genetica Molecular and Subdepartamento de Virologia ISP Chile	Instituto de Salud Publica de Chile	Andres Castillo; Barbara Parra; Constanza Campano; Gisselle Barra; Javier Tognarelli; Jorge Fernandez; Karen Orostica; Loredana Arata; Patricia Bustos; Rodrigo Fasce; Soledad Ulloa
EPI_ISL_2301853	Greek Genome Center, Biomedical Research Foundation of the Academy of Athens (BRFAA)	Greek Genome Center, Biomedical Research Foundation of the Academy of Athens (BRFAA)	Dimitrios Thanos; Emmanouil Athanasiadis; Giannis Vatsellas; Katerina Zoi; Theodoros Loupis
EPI_ISL_2187692	HLAGYN - Laboratorio de Imunologia de Transplantes de Goias	HLAGYN - Laboratorio de Imunologia de Transplantes de Goias	Alessandro Leonardo Alvares Magalhaes; Daniel Ferreira de Sousa; Erika Lopes Rocha Batista; Fernando Antonio Vinhal dos Santos; Frederico Rodrigues Vinhal; Lucas Carlos Gomes Pereira; Sabrina Sara Moreira Duarte
EPI_ISL_1581735, EPI_ISL_1907121, EPI_ISL_4346271, EPI_ISL_4962483	Helix/Illumina	Centers for Disease Control and Prevention Division of Viral Diseases, Pathogen Discovery	Adrian Paskey; Alexandre Bolze; Ary Ascencio; Benjamin Rambo-Martin; Brad Sickler; Charlotte Rivera-Garcia; Christine Tran; Christopher Gulvick; Chrstine Tran; Clinton Paden; Clinton R. Paden; Dakota Howard; Darlene Wagner; David Becker; Dhwani Batra; Duncan MacCannell; Efren Sandoval; Eileen De Feo; Eileen de Feo; Elizabeth Cirulli; Eric Allen; Geraint Levan; James Lu; Jan Antico; Jason Caravas; Jason Nguyen; Jimmy Ramirez; Jingtao Liu; Kara Moser; Kelly Barrett; Kelly Schiabor Barrett; Kim Gietzen; Kristine Lacek; Magnus Isaksson; Marc Laurent; Matthew Schmerer; Matthew Tolentino; Nicole L. Washington; Nicole Washington; Peter Cook; Peter W. Cook; Phil Febbo; Ryan Cho; Scott Sammons; Shannon Wickline; Shatavia Morrison; Sherry Wang; Simon White; Tyler Cassens; William Lee; Yvette Unoarumhi
EPI_ISL_5070788	Houston Methodist Hospital	Houston Methodist Hospital	Ilya J. Finkelstein; James J. Davis; Jessica Cambric; Jimmy Gollihar; Kristina Reppond; Layne Pruitt; Madison N. Shyer; Marcus Nguyen; Matthew Ojeda Saavedra; Paul A. Christensen; Prasanti Yerramilli; Randall J. Olsen; Robert Olson; Ryan Gadd; S. Wesley Long; Sishir Subedi; and James M. Musser
EPI_ISL_2152548	Hôpital Cochin	Department of Virology, Henri Mondor University Hospital, Assistance Publique Hôpitaux de Paris, Université Paris-Est Créteil, INSERM U955	Alexandre Soulier; Christophe Rodriguez; Elisabeth Trawinski; Guillaume Gricourt; Jean-Michel Pawlotsky; Melissa N'Debi; Slim Fourati; Vanessa Demontant
EPI_ISL_1819243	I.R.C.C.S. "S. De Bellis" - Ente Ospedaliero	Istituto Zooprofilattico Sperimentale della Puglia e della Basilicata	Bianco A.; Capozzi L.; Del Sambro L.; Lippolis A.; Notarnicola M.; Parisi A.; Simone D.
EPI_ISL_1678325, EPI_ISL_1678326	ID Bureau of Laboratories	Centers for Disease Control and Prevention Division of Viral Diseases, Pathogen Discovery	Alison Laufer Halpin; Ben L. Rambo-Martin; Clinton R. Paden; Dakota Howard; Darlene Wagner; Dave Wentworth; Dhwani Batra; Jasmine Padilla; Justin Lee; Katie Dillon; Krista Queen; Kristen Knipe; Kristine Lacek; Mark Burroughs; Matthew Schmerer; Mili Sheth; Peter Cook; Sam Shepard; Sarah Nobles; Shoshona Le; Suxiang Tong; Vivien Dugan; Yvette Unoarumhi
EPI_ISL_3275089, EPI_ISL_3275095	Idaho Bureau of Laboratories	Center for Global Health, University of New Mexico Health Sciences Center	Christopher Ball; Darrell Dinwiddie; Daryl Domman; Kurt Schwalm; Matthew Burns; Robert Voermans; Valerie Morley
EPI_ISL_1219920, EPI_ISL_3543382, EPI_ISL_3756841, EPI_ISL_3922840, EPI_ISL_3923187	Idaho Bureau of Laboratories	Idaho Bureau of Laboratories	"R. Beukelman; Aimee Ceniseros; Christian Loera; Christopher Ball"; Matthew Charles Burns; Robert L. Voermans
EPI_ISL_1964532, EPI_ISL_3039233, EPI_ISL_3039248	Institute of Microbiology and Immunology, Faculty of Medicine, University of Ljubljana	Institute of Microbiology and Immunology, Faculty of Medicine, University of Ljubljana	Alen Suljič; Andraž Celar; Dominika Šturm; Doroteja Vlaj; Mario Poljak; Matic Brvar; Miša Korva; Patricija Pozvek; Samo Zakotnik; Tatjana Avšič – Županc; Tomaž Mark Zorec; Špela Pleh
EPI_ISL_4862793	Integrated Covid Hub North East	Wellcome Sanger Institute for the COVID-19 Genomics UK (COG-UK) Consortium	Cordelia Langford; David K. Jackson; Dominic Kwiatkowski; Ewan Harrison; Ian Johnston; Integrated Covid Hub North East and Alex Alderton; Jeffrey Barrett; John Sillitoe on behalf of the Wellcome Sanger Institute COVID-19 Surveillance Team; Roberto Amato; Sonia Goncalves
EPI_ISL_1689555	Lab voor klinische biologie	Lab voor klinische biologie	Bruno Verhasselt; Hannelore Hamerlinck; Marija Janevska

EPI_ISL_1140857, EPI_ISL_1141807	Labor Dr. Wisplinghoff - Berlin	Robert Koch Institute	
EPI_ISL_1643477	Labor LÃ¼beck bzw. LaborÃ¤rztliche Gemeinschaftspraxis LÃ¼beck	Robert Koch Institute	
EPI_ISL_1218124, EPI_ISL_1282942	Labor ZOTZ|KLIMAS; MVZ Düsseldorf-Centrum	Robert Koch Institute	
EPI_ISL_5617461	Laboratorio Nacional de Salud, Ministerio de Salud Publica y Asistencia Social	Genomics and Proteomics Departament, Gorgas Memorial Institute For Health Studies	Alexander A Martinez; Ambar Moreno; Claudia Estrada; Claudia Gonzalez V; César Roberto Conde Pereira; Jessica Gondola; Leyda Abrego; Marlene Castillo; Melissa Gaitan; Oris Chavarria
EPI_ISL_4635252, EPI_ISL_5504867	Laboratorio de Referencia Nacional de Virus Respiratorios. Centro Nacional de Salud Publica. Instituto Nacional de Salud Peru.	Laboratorio de Referencia Nacional de Virus Respiratorios. Centro Nacional de Salud Publica. Instituto Nacional de Salud Peru.	Alicia Nuñez Llanos; Carlos Padilla Rojas; Henri Bailon Calderon; Iris Silva Molina; Joseph Huayra Niquen; Lely Solari Zerpa; Luis Barcena Flores; Marco Galarza Perez; Nancy Rojas Serrano; Nieves Sevilla Castañeda; Omar Caceres Rey; Orson Mestanza Millones; Princesa Medrano Alhuay; Priscila Lope Pari; Sandra Morales Ruiz; Sara Gordillo Vilchez; Steve Acedo Lazo; Veronica Hurtado Vela; Victor Jimenez Vasquez; Wendy Lizarraga Olivares
EPI_ISL_2044785, EPI_ISL_2927081	Laboratory Corporation of America	Centers for Disease Control and Prevention Division of Viral Diseases, Pathogen Discovery	Adrian Paskey; Amanda Douglas; Amanda Suchanek; Andrea Throop; Ayla Burns; Benjamin Rambo-Martin; Bobbi Croy; Brian Krueger; Brian Norvell; Christopher Gulvick; Christos Petropoulos; Clinton R. Paden; Craig Lukasik; Dakota Howard; Darlene Wagner; Debbie Boles; Dhwani Batra; Duncan MacCannell; Eyad Almasri; Goran Stevovic; Howard Engler; Hrushikesh Deshmukh; Jake Humphrey; Jana Schroth; Jason Caravas; Joe Voshell; John Pruitt; Jonathan Meltzer; Jonathan Williams; Kara Moser; Kimberly Wagner; Lax Iyer; Lisa Pfefferle; Lyndon Tilson; Manoj Jain; Marcia Eisenberg; Mary Ann Cristobal; Mary Williamson; Matthew Robinson; Matthew Schmerer; Michael Levandoski; Mike Sapeta; Mindy Nye; Minoo Agarwal; Mohan Kolli; Nuthawin Charoensri; Oren Cohen; Peter W. Cook; Prashant Gupta; Qian Zeng; Rama Ghatti; Scott Parker; Scott Ryan; Scott Sammons; Shatavia Morrison; Stanley Letovsky; Steven Ragan; Suresh Babu Selvaraju; Susan Countryman; Susan Hicks; Suzanne Dale; Thomas Urban; Tim Kuphal; Tricia Zwiefelhofer; Vincent Drouillon; Yvette Unoarumhi
EPI_ISL_1504634, EPI_ISL_2393531, EPI_ISL_2569439, EPI_ISL_4099792, EPI_ISL_4189290, EPI_ISL_4191032, EPI_ISL_4754011			
see above	Lighthouse Lab in Alderley Park	Wellcome Sanger Institute for the COVID-19 Genomics UK (COG-UK) Consortium	Cordelia Langford; David K. Jackson; Dominic Kwiatkowski; Ewan Harrison; Ian Johnston; Jacquelyn Wynn; Jeffrey Barrett; John Sillitoe on behalf of the Wellcome Sanger Institute COVID-19 Surveillance Team; Mairead Hyland; Roberto Amato; Sonia Goncalves; The Lighthouse Lab in Alderley Park and Alex Alderton
EPI_ISL_822325, EPI_ISL_2513334, EPI_ISL_4289982, EPI_ISL_4683454	Lighthouse Lab in Glasgow	Wellcome Sanger Institute for the COVID-19 Genomics UK (COG-UK) Consortium	Anna Dominiczak and Alex Alderton; Carol Clugston; Cordelia Langford; David Gray; David K. Jackson; Dominic Kwiatkowski; Ewan Harrison; Harper VanSteenhouse; Ian Johnston; Jeffrey Barrett; John Sillitoe on behalf of the Wellcome Sanger Institute COVID-19 Surveillance Team; Roberto Amato; Sonia Goncalves; Yumi Kasai
EPI_ISL_993421, EPI_ISL_2118914, EPI_ISL_2236023, EPI_ISL_3763330	Lighthouse Lab in Milton Keynes	Wellcome Sanger Institute for the COVID-19 Genomics UK (COG-UK) Consortium	Cordelia Langford; David K. Jackson; Dominic Kwiatkowski; Ewan Harrison; Ian Johnston; Jeffrey Barrett; John Sillitoe on behalf of the Wellcome Sanger Institute COVID-19 Surveillance Team; Roberto Amato; Sonia Goncalves; The Lighthouse Lab in Milton Keynes and Alex Alderton
EPI_ISL_1280209	Limbach - MVZ Labor Eveld & Kollegen Essen	Robert Koch Institute	
EPI_ISL_1153475	MVZ Dr. Eberhard & Partner Dortmund	Robert Koch Institute	
EPI_ISL_1218607, EPI_ISL_1439643, EPI_ISL_1439663, EPI_ISL_1439670, EPI_ISL_1440873, EPI_ISL_1440888, EPI_ISL_1440919			
see above	MVZ Labor Krone GbR	Robert Koch Institute	
EPI_ISL_1347837	Medical Laboratories Duesseldorf	Center of Medical Microbiology, Virology, and Hospital Hygiene, University of Duesseldorf	Alexander Dilthey; Andreas Walker; Angelika Helmer; Christian Lange; Daniel Strelow; Jessica Nicolai; Jörg Timm; Klaus Pfeffer; Lisanna Hülse; Malte Kohns Vasconcelos; Maximilian Damagnez; Nadine Lübke; Tobias Wienemann; Torsten Houwaart
EPI_ISL_1527767	Michigan Department of Health and Human Services, Bureau of Laboratories	Michigan Department of Health and Human Services, Bureau of Laboratories	Blankenship HM; Riner D; Soehnlen MK
EPI_ISL_5015439, EPI_ISL_5015483	Nastavni zavod za javno zdravstvo Splitsko- Dalmatinske županije	Hrvatski zavod za javno zdravstvo	Irena Tabain; Ivana Ferenčak
EPI_ISL_1915533	National Institute of Health Research and Development	National Institute of Health Research and Development	Agustiningsih; Arie Ardiansyah Nugraha; Fauzul Muna; Hana Apsari Pawestri; Hartanti Dian Ikawati; Herna; Holy Arif Wibowo; Irene Lorinda Indalao; Kartika Dewi Puspa; Kindi Adam; Krisna Nur Andriana Pangesti; Natalie Laurencia Kipuw; Nelly Puspandari; Ni Ketut Susilarini; Nike Susanti; Nurika Hariastuti; Reni Herman; Ririn Ramadhany; Subangkit; Tati Febriyanti; Triyani Soekarso; Ully Alfi Nikmah; Vivi Setiawaty.; Yuni Rukminiati
EPI_ISL_1911219	Naval Infectious Diseases Diagnostic Laboratory	Naval Medical Research Center Biological Defense Research Directorate	Andrea Luquette; Andrew Bennett; Bishwo Adhikari; Catherine Arnold; Francisco Malagon Bautista; Gregory Rice; Kimberly Bishop-Lilly; Kyle Long; Lindsay Glang; Logan Voegtly; Megan Schilling; Michael Deschenes; Regina Cer; Victor Sugiharto
EPI_ISL_1196461	New Mexico Department of Health Scientific Laboratory	New Mexico Department of Health Scientific Laboratory	Anastacia Griego-Fisher; D'eldra Malone; Ellie Johnson; Jennifer Benoit
EPI_ISL_3903716	Nicolae Testemitanu State University of Medicine and Pharmacy	International Centre for Genetic Engineering and Biotechnology (ICGEB) and ARGO Open Lab Platform for Genome Sequencing	Buzurnii L; Dal Monego S; Groppa S; Licastro D; Marcello A; Orsini E; Paraschiv A; Todiras M; Ulinici M
EPI_ISL_612110	Northumbria University / South Tees Hospitals NHS Foundation Trust / North Cumbria Integrated Care NHS Foundation Trust / North Tees and Hartlepool NHS Foundation Trust / Newcastle Hospitals NHS Foundation Trust	COVID-19 Genomics UK (COG-UK) Consortium	Andrew Nelson; Brendan Payne; Clive Graham; Darren L Smith; Debra Padgett; Edward Barton; Emma Swindells; Garren Scott; Gary Black; Gary Eltringham; Giles S Holt; Greg R Young; Jane Greenaway; Jennifer Collins; John Allan; Joshua Loh; Lynn Dover; Matthew Bashton; Mohammad A Tariq; Paul Baker; Sarah Essex; Steve Liggett; Wen C Yew; Yusri Taha
EPI_ISL_2373016	Northwestern Memorial Hospital	Northwestern University - Ozer Lab	Chad J. Achenbach; Chao Qi; Egon A. Ozer; Judd F. Hultquist; Lacy M. Simons; Lawrence J. Jennings; Michael G. Ison; Ramon Lorenzo-Redondo; Taylor J. Dean
EPI_ISL_5294882	Originating lab: Wales Specialist Virology Centre Sequencing lab: Pathogen Genomics Unit	Public Health Wales Microbiology Cardiff Wales Specialist Virology Centre	Alec Birchley; Alexander Adams; Amy Gaskin; Angela Marchbank; Bree Gatica-Wilcox; Catherine Moore; Jason Coombes; Joanne Watkins; Joel Southgate; Johnathan Evans; Laura Gifford; Lauren Gilbert; Lee Graham; Malorie Perry; Matthew Bull; Nicole Pacchiarini; Sally Corden; Sara Kumziene-Summerhayes; Sara Rey; Sarah Taylor; Simon Cottrell; Sophie Jones; Tom Connor
EPI_ISL_2037330	Pandemic Response Lab - NYC	Pandemic Response Lab, R&D	Cybill del Castillo; Dylan Law; Haiping Hao; Henry Lee; Jon Laurent; Katharine Nelson; Melissa Hopkins; Michael Hammerling; Pradeep Bugga; Shinyoung Clair Kang; Sol Rey; William Ward
EPI_ISL_1281983, EPI_ISL_1435362	Procomcure Biotech Germany GmbH	Robert Koch Institute	
EPI_ISL_2253727	Randox Laboratories	Wellcome Sanger Institute for the COVID-19 Genomics UK (COG-UK) Consortium	Cordelia Langford; David K. Jackson; Dominic Kwiatkowski; Ewan Harrison; Ian Johnston; Jeffrey Barrett; John Sillitoe on behalf of the Wellcome Sanger Institute COVID-19 Surveillance Team; Randox Laboratories and Alex Alderton; Roberto Amato; Sonia Goncalves
EPI_ISL_5041500, EPI_ISL_5281579	Respiratory Virus Unit, Microbiology Services Colindale, Public Health England	COVID-19 Genomics UK (COG-UK) Consortium	PHE Covid Sequencing Team
EPI_ISL_2776550	SARS-CoV-2 testing team, National Institute of Infectious Diseases	Pathogen Genomics Center, National Institute of Infectious Diseases	Hazuka Y Furihata; Hiromizu Takahashi; Kentaro Itokawa; Makoto Kuroda; Masanori Hashino; Masumichi Saito; Naomi Nojiri; Nozomu Hanaoka; Rina Tanaka; Tsuguto Fujimoto; Tsuyoshi Sekizuka
EPI_ISL_2112492	SYNLAB Jena Oncoscreen	Robert Koch Institute	
EPI_ISL_1729353, EPI_ISL_1851074, EPI_ISL_2110293	SYNLAB MVZ Berlin	Robert Koch Institute	
EPI_ISL_2130345	SYNLAB MVZ Leverkusen	Robert Koch Institute	
EPI_ISL_2934764, EPI_ISL_2934792, EPI_ISL_2934796	Servicio de Microbiología Clínica (Complejo Hospitalario de Navarra, Pamplona)	Centro de Secuenciación NASERTIC	Ana Miqueleiz; Ana Navascués; Carmen Ezpeleta Baquedano
EPI_ISL_2383298	Servicio de Microbiología. Hospital General	SeqCOVID-SPAIN	María Dolores Tirado Balaguer and SeqCOVID-SPAIN consortium; Rosario Moreno Muñoz

	Universitario de Castellón	consortium/IBV(CSIC)	
EPI_ISL_4518949, EPI_ISL_4519084	Sharp HealthCare Laboratory	Andersen lab at Scripps Research	Art Mendoza; Cathy Woerle; Jacquelyn Berumen; Liam McGinnis; Omid Bakhtar; SEARCH Alliance San Diego with Aaron Harding
EPI_ISL_1770548	State Laboratories Division, Hawaii State Department of Health	State Laboratories Division, Hawaii State Department of Health	Ayana Garnet; Drew Kuwazaki; Edward Desmond; Pamela O'Brien; Razvan Sultana
EPI_ISL_1899466, EPI_ISL_2419503	Swedish national genomic surveillance program of SARS-CoV-2	The Public Health Agency of Sweden	Alma Brolund; Maria Lind Karlberg; Maximilian Riess; Swedish national genomic surveillance program of SARS-CoV-2
EPI_ISL_3655956	University of Florida Health Pathology Laboratories	Salemi Lab, University of Florida	Cash MN; Lauzardo M; Magalis BR; Mavian C; Riva A; Salemi M; Tagliamonte M
EPI_ISL_2292512	Utah Public Health Laboratory	Utah Public Health Laboratory	Erin L. Young; Kelly F. Oakeson; Tara Gallagher
EPI_ISL_456551	Victorian Infectious Diseases Reference Laboratory (VIDRL)	Microbiological Diagnostic Unit Public Health Laboratory and Victorian Infectious Diseases Reference Laboratory, Doherty Institute	Caly L.; Druce J.; Sait, M.; Schultz M.; Seemann T.; Sherry, N.
EPI_ISL_2662416	Viollier AG	Department of Biosystems Science and Engineering, ETH Zürich	Andrea Patrizia Salzmann; Chaoran Chen; Christiane Beckmann; Christoph Noppen; Henriette Kurth; Ivan Topolsky; Kim Philipp Jablonski; Lara Fuhrmann; Maurice Redondo; Niko Beerenwinkel; Olivier Kobel; Sarah Nadeau; Tanja Stadler
EPI_ISL_3115359	Virology laboratory, University of Maiduguri Teaching Hospital	Africa Centre for Excellence for Genomics of Infectious Diseases (ACEGID), Redeemer's University	A.T.; Abechi; Ajogbasile; Akano; C.A.; C.T.; Eromon; F.V.; Folarin, O.; Happi; I.B.; J.N.; J.U.; K.O.; Kayode; Nosamiefan, I.; Oguzie; Olawoye; Olumade; Oluniyi; P.E.; P.S.; T.J.; Ugwu; Uwanibe
EPI_ISL_473537	West of Scotland Specialist Virology Centre, NHSGGC / MRC-University of Glasgow Centre for Virus Research	COVID-19 Genomics UK (COG-UK) Consortium	Alasdair MacLean; Alice Broos; Ana da Silva Filipe; Antonia Ho; Daniel Mair; David L Robertson; Elihu Aranday-Cortes; Emma Thomson; James Shepherd; Jenna Nichols; Joseph Hughes; Kathy Li; Kathy Smollett; Kirstyn Brunker; Kyriaki Nomikou; Lily Tong; Marc Niebel; Natasha Jesudason; Natasha Johnson; Patawee Asamaphan; Rajiv Shah; Richard Orton; Rory Gunson; Sarah McDonald; Sreenu Vattipally; Stephen Carmichael; Yasmin Parr
EPI_ISL_1447611, EPI_ISL_2296455	Yale Clinical Virology Lab	Grubaugh Lab - Yale School of Public Health	Anderson Brito; Annie Watkins; Chaney Kalinich; Chantal Vogels; Isabel Ott; Jessica Rothman; Joseph Fauver; Mallery Breban; Marie L. Landry; Mary Petrone; Nathan Grubaugh; Tara Alpert
EPI_ISL_1727175	amedes MVZ Hannover	Robert Koch Institute	
EPI_ISL_1156939	amedes MVZ für Laboratoriumsmedizin Rhein/Ruhr	Robert Koch Institute	

Supplementary Table 4: GISAID acknowledgments for genomes containing nsp12 E802D used to generate Supplementary Figure 3
