# Supplementary material for: De novo emergence of a remdesivir resistance mutation during treatment of persistent SARS-CoV-2 infection in an immunocompromised patient: a case report
Source: Nat Commun. 2022 Mar 17;13:1547. doi: 10.1038/s41467-022-29104-y (PMC8930970; doi:10.1038/s41467-022-29104-y)
Supplement: Supplementary file 3 — Reporting Summary [file 41467_2022_29104_MOESM3_ESM.pdf]

nature portfolio

Corresponding author(s): Shiv Gandhi
Albert Ko

Last updated by author(s): Feb 23, 2022

Reporting Summary

Nature Portfolio wishes to improve the reproducibility of the work that we publish. This form provides structure for consistency and transparency in reporting. For further information on Nature Portfolio policies, see our Editorial Policies and the Editorial Policy Checklist.

Statistics

For all statistical analyses, confirm that the following items are present in the figure legend, table legend, main text, or Methods section.

n/a	Confirmed	
☐	☒	The exact sample size (*n*) for each experimental group/condition, given as a discrete number and unit of measurement
☒	☐	A statement on whether measurements were taken from distinct samples or whether the same sample was measured repeatedly
☐	☒	The statistical test(s) used AND whether they are one- or two-sided<br>*Only common tests should be described solely by name; describe more complex techniques in the Methods section.*
☒	☐	A description of all covariates tested
☒	☐	A description of any assumptions or corrections, such as tests of normality and adjustment for multiple comparisons
☐	☒	A full description of the statistical parameters including central tendency (e.g. means) or other basic estimates (e.g. regression coefficient) AND variation (e.g. standard deviation) or associated estimates of uncertainty (e.g. confidence intervals)
☐	☒	For null hypothesis testing, the test statistic (e.g. *F*, *t*, *r*) with confidence intervals, effect sizes, degrees of freedom and *P* value noted<br>*Give P values as exact values whenever suitable.*
☒	☐	For Bayesian analysis, information on the choice of priors and Markov chain Monte Carlo settings
☒	☐	For hierarchical and complex designs, identification of the appropriate level for tests and full reporting of outcomes
☒	☐	Estimates of effect sizes (e.g. Cohen's *d*, Pearson's *r*), indicating how they were calculated

Our web collection on statistics for biologists contains articles on many of the points above.

Software and code

Policy information about availability of computer code

Data collection	No software used to collect data.
Data analysis	Phylogenetic trees were constructed with Nextstrain package (Version 3.0.6) using data downloaded from GISAID. Clinical and virological data was plotted in the R environment using RStudio (Version 1.3.1093). Biological experiments were plotted with Graphpad Prism (Version 9.3.1). Sequencing reads were processed in a custom bioinformatics pipeline (github link in manuscript). Flow cytometry analyzed with FlowJo 10.6 (TreeStar). PAML (version 4.8) used for positive selection analysis.

For manuscripts utilizing custom algorithms or software that are central to the research but not yet described in published literature, software must be made available to editors and reviewers. We strongly encourage code deposition in a community repository (e.g. GitHub). See the Nature Portfolio guidelines for submitting code & software for further information.

Data

Policy information about availability of data

All manuscripts must include a data availability statement. This statement should provide the following information, where applicable:

- Accession codes, unique identifiers, or web links for publicly available datasets
- A description of any restrictions on data availability
- For clinical datasets or third party data, please ensure that the statement adheres to our policy

Sequencing data are available under NCBI BioProject no. PRJNA774781 (Supplementary Table 2).

Field-specific reporting

Please select the one below that is the best fit for your research. If you are not sure, read the appropriate sections before making your selection.

- [x] Life sciences
- [ ] Behavioural & social sciences
- [ ] Ecological, evolutionary & environmental sciences

For a reference copy of the document with all sections, see nature.com/documents/nr-reporting-summary-flat.pdf

Life sciences study design

All studies must disclose on these points even when the disclosure is negative.

Sample size	The clinical component is a case study of one patient. Experimental work included at least three biological replicates which was sufficient to find statistically significant changes at the expected effect size of treatment (ie RDV).
Data exclusions	No data was excluded from this paper
Replication	All experiments depicted included at least three biological replicates and were representative of at least 2 experimental replicates
Randomization	Randomization was not applicable as this is a case study of a single individual
Blinding	No blinding was performed as this was a case study of a single individual and the end points of the experiments were objective measures and not subject to subjective interpretation.

Reporting for specific materials, systems and methods

We require information from authors about some types of materials, experimental systems and methods used in many studies. Here, indicate whether each material, system or method listed is relevant to your study. If you are not sure if a list item applies to your research, read the appropriate section before selecting a response.

Materials & experimental systems

n/a	Involved in the study	
[ ]	[x]	Antibodies
[ ]	[x]	Eukaryotic cell lines
[x]	[ ]	Palaeontology and archaeology
[x]	[ ]	Animals and other organisms
[ ]	[x]	Human research participants
[ ]	[x]	Clinical data
[x]	[ ]	Dual use research of concern

Methods

n/a	Involved in the study	
[x]	[ ]	ChIP-seq
[ ]	[x]	Flow cytometry
[x]	[ ]	MRI-based neuroimaging

Antibodies

Antibodies used	All antibodies used in flow cytomertry are against human proteins. BB515 anti-hHLA-DR (G46-6) (1:400) (BD Biosciences), BV605 anti-hCD3 (UCHT1) (1:300) (BioLegend), BV785 anti-hCD4 (SK3) (1:200) (BioLegend), APCFire750 or PE-Cy7 or BV711 anti-hCD8 (SK1) (1:200) (BioLegend), BV421 anti-hCCR7 (G043H7) (1:50) (BioLegend), AlexaFluor 700 anti-hCD45RA (HI100) (1:200) (BD Biosciences), PE anti-hPD1 (EH12.2H7) (1:200) (BioLegend), APC anti-hTIM3 (F38-2E2) (1:50) (BioLegend), BV711 anti-hCD38 (HIT2) (1:200) (BioLegend), BB700 anti-hCXCR5 (RF8B2) (1:50) (BD Biosciences), PECy7 anti-hCD127 (HIL-7R-M21) (1:50) (BioLegend), PE-CF594 anti-hCD25 (BC96) (1:200) (BD Biosciences).
Validation	All antibodies used in flow cytometry study are commercially available, and all have been validated by the manufacturers and used by other publications. Likewise, we titrated these antibodies according to our own our staining conditions. The following were validated in the following species: BB515 anti-hHLA-DR (G46-6) (BD Biosciences) (Human, Rhesus, Cynomolgus, Baboon), BV605 anti-hCD3 (UCHT1) (BioLegend) (Human, Chimpanzee), BV785 anti-hCD4 (SK3) (BioLegend) (Human), APCFire750 or PE-Cy7 or BV711 anti-hCD8 (SK1) (BioLegend) (Human, Cross-Reactivity: African Green, Chimpanzee, Cynomolgus, Pigtailed Macaque, Rhesus, Sooty Mangabey), BV421 anti-hCCR7 (G043H7) (BioLegend) (Human, African Green, Baboon, Cynomolgus, Rhesus), AlexaFluor 700 anti-hCD45RA (HI100) (BD Biosciences) (Human), PE anti-hPD1 (EH12.2H7) (BioLegend) (Human, African Green, Baboon, Chimpanzee, Common Marmoset, Cynomolgus, Rhesus, Squirrel Monkey), APC antihTIM3 (F38-2E2) (BioLegend) (Human), BV711 anti-hCD38 (HIT2) (BioLegend) (Human, Chimpanzee, Horse), BB700 anti-hCXCR5 (RF8B2) (BD Biosciences) (Human), PE-Cy7 anti-hCD127 (HIL-7R-M21) (BioLegend) (Human), PE-CF594 anti-hCD25 (BC96) (BD Biosciences) (Human, Rhesus, Cynomolgus, Baboon).

Eukaryotic cell lines

Policy information about cell lines

Cell line source(s)	Vero E6 cells obtained from ATCC
Authentication	Cell lines were not authenticated
Mycoplasma contamination	Cell lines were tested negative for mycoplasma by PCR
Commonly misidentified lines (See ICLAC register)	No commonly misidentified cell lines were used in this study - Vero cells are not as a mistaken cell line in V11 of the ICLAC register

Human research participants

Policy information about studies involving human research participants

Population characteristics	Single case of a 70-year-old female
Recruitment	This was a single patient who was referred to our institution for management of persistent SARS-CoV-2 infection. Given that our findings are limited to a single individual, they will need to be confirmed in a broader population.
Ethics oversight	Yale Human Research Protection Program Institutional Review Boards (FWA00002571, protocol ID 2000027690, 2000029277)

Note that full information on the approval of the study protocol must also be provided in the manuscript.

Clinical data

Policy information about clinical studies

All manuscripts should comply with the ICMJEguidelines for publication of clinical research and a completedCONSORT checklist must be included with all submissions.

Clinical trial registration	*Provide the trial registration number from ClinicalTrials.gov or an equivalent agency.*
Study protocol	*Note where the full trial protocol can be accessed OR if not available, explain why.*
Data collection	*Describe the settings and locales of data collection, noting the time periods of recruitment and data collection.*
Outcomes	*Describe how you pre-defined primary and secondary outcome measures and how you assessed these measures.*

Flow Cytometry

Plots

Confirm that:

- [x] The axis labels state the marker and fluorochrome used (e.g. CD4-FITC).
- [x] The axis scales are clearly visible. Include numbers along axes only for bottom left plot of group (a 'group' is an analysis of identical markers).
- [x] All plots are contour plots with outliers or pseudocolor plots.
- [x] A numerical value for number of cells or percentage (with statistics) is provided.

Methodology

Sample preparation	PBMC isolated via density centrifugation (see methods for full details). After plating on a 96-well plate, cells were resuspended in live/dead fixation and then blocked with Trustan FcX. Antibodies were then added for 30m at RT. Wells were then washed and fixed in PFA.
Instrument	Attune NXT (ThermoFisher)
Software	FlowJo
Cell population abundance	Cell population abundance: Cells populations were reported in various formats including as a number or concentration of the patient's blood sample (x106cells/mL), as a proportion of live, single PBMC (% of Live), or as a proportion of a parent gate (% of CD4 T cells, % of CD8 T cells, etc.). The full gating path for clarification is included in Figure S8.

Gating strategy	SSC-A and FSC-A parameters were used to select lymphocytes from isolated PBMCs. Live and dead cells were defined based on aqua staining. Singlets were separated based on SSC/ FSC parameters. Lymphocytess were gated based on to identify T lymphocytes (CD3/CD4/CD8 markers). TCR-activated T cells, Terminally-differentiated T cells, and additional subsets were defined using HLA-DR, CD38, CCR7,CD127, PD1, TIM-3, CXCR5, CD45RA, CD25.

☒ Tick this box to confirm that a figure exemplifying the gating strategy is provided in the Supplementary Information.
